# Cover crop monocultures and mixtures enhance bacterial abundance and functionality in the maize root zone

**DOI:** 10.1093/ismeco/ycae132

**Published:** 2024-10-29

**Authors:** Debjyoti Ghosh, Yijie Shi, Iris M Zimmermann, Tobias Stürzebecher, Katja Holzhauser, Martin von Bergen, Anne-Kristin Kaster, Sandra Spielvogel, Michaela A Dippold, Jochen A Müller, Nico Jehmlich

**Affiliations:** Department of Molecular Toxicology, Helmholtz Centre for Environmental Research (UFZ), Permoserstraße 15, 04318 Leipzig, Saxony, Germany; Institute of Plant Nutrition and Soil Science, Department of Soil Science, Christian-Albrechts-University Kiel, Hermann-Rodewald-Straße 2, 24118 Kiel, Schleswig-Holstein, Germany; Institute of Plant Nutrition and Soil Science, Department of Soil Science, Christian-Albrechts-University Kiel, Hermann-Rodewald-Straße 2, 24118 Kiel, Schleswig-Holstein, Germany; Biogeochemistry of Agroecosystems, University of Göttingen, Büsgenweg 2, 37077 Göttingen, Lower Saxony, Germany; Institute of Crop Science and Plant Breeding, Agronomy and Crop Science, Christian-Albrechts-University Kiel, Am Botanischen Garten 1-9, 24118 Kiel, Schleswig-Holstein, Germany; Department of Molecular Toxicology, Helmholtz Centre for Environmental Research (UFZ), Permoserstraße 15, 04318 Leipzig, Saxony, Germany; Institute for Biochemistry, Faculty of Biosciences, Pharmacy and Psychology, University of Leipzig, Brüderstraße 34, 04103 Leipzig, Saxony, Germany; German Centre for Integrative Biodiversity Research (iDiv) Halle-Jena-Leipzig, Puschstraße 4, 04103 Leipzig, Saxony, Germany; Institute for Biological Interfaces, Karlsruhe Institute of Technology, Hermann-von-Helmholtz-Platz 1, 76344 Eggenstein-Leopoldshafen, Baden-Württemberg, Germany; Institute of Plant Nutrition and Soil Science, Department of Soil Science, Christian-Albrechts-University Kiel, Hermann-Rodewald-Straße 2, 24118 Kiel, Schleswig-Holstein, Germany; Geo-Biosphere Interactions, Department of Geosciences, University of Tübingen, Schnarrenbergstraße 94-96, 72076 Tübingen, Baden-Württemberg, Germany; Institute for Biological Interfaces, Karlsruhe Institute of Technology, Hermann-von-Helmholtz-Platz 1, 76344 Eggenstein-Leopoldshafen, Baden-Württemberg, Germany; Department of Molecular Toxicology, Helmholtz Centre for Environmental Research (UFZ), Permoserstraße 15, 04318 Leipzig, Saxony, Germany

**Keywords:** cover crop, root channel reuse, bacterial community, rhizosphere, C cycle, N cycle, metaproteomics

## Abstract

Cover cropping is an effective method to protect agricultural soils from erosion, promote nutrient and moisture retention, encourage beneficial microbial activity, and maintain soil structure. Re-utilization of winter cover crop root channels by maize roots during summer allows the cash crop to extract resources from distal regions in the soil horizon. In this study, we investigated how cover cropping during winter followed by maize (*Zea mays* L.) during summer affects the spatiotemporal composition and function of the bacterial communities in the maize rhizosphere and surrounding soil samples using quantitative polymerase chain reaction (PCR), 16S ribosomal ribonucleic acid (rRNA) gene amplicon sequencing, and metaproteomics. We found that the bacterial community differed significantly among cover crop species, soil depths, and maize growth stages. Bacterial abundance increased in reused root channels, and it continued to increase as cover crop diversity changed from monocultures to mixtures. Mixing *Fabaceae* with *Brassicaceae* or *Poaceae* enhanced the overall contributions of several steps of the bacterial carbon and nitrogen cycles, especially glycolysis and the pentose phosphate pathway. The deeper root channels of *Fabaceae* and *Brassicaceae* as compared to *Poaceae* corresponded to higher bacterial 16S rRNA gene copy numbers and improved community presence in the subsoil regimes, likely due to the increased availability of root exudates secreted by maize roots. In conclusion, root channel reuse improved the expression of metabolic pathways of the carbon and nitrogen cycles and the bacterial communities, which is beneficial to the soil and to the growing crops.

## Graphical Abstract

**Graphical Abstract:**
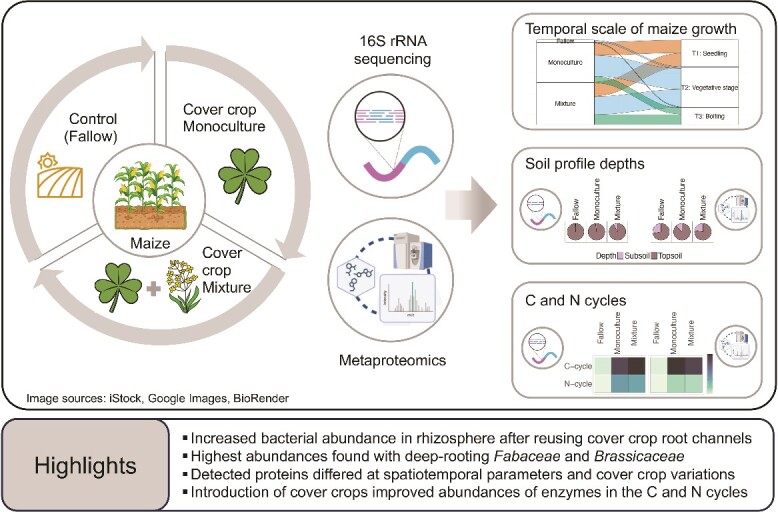

## Introduction

Anthropogenic climate change is having a major impact on agriculture in many regions of the world due to shifts in precipitation patterns and accelerated carbon (C) loss from soils [1, 2]. Altered precipitation patterns pose a double jeopardy, as topsoil desiccation during droughts limits crop productivity, while heavy rainstorms cause erosion of fallow land. Cover cropping can play an effective role in agricultural soil and water management by improving erosion protection, water infiltration, and moisture retention. Additional benefits of cover cropping include increased soil C and nitrogen (N) storage and mitigation of nitrate leaching [3–8].

There are still substantial knowledge gaps on cover cropping, particularly whether and how the cash crop benefits from the soil imprints of the preceding cover crop. The ease and improvement of crop root access to subsoil water and nutrient reserves, in addition to soil hardness and temperature, are still understudied [9]. Pre-existing plant roots create root channels that reduce penetration resistance and promote root growth into deeper soil [10], depending upon different soil types. When the cover crop dies back, channels from their roots remaining in the soil can be reused by the subsequent cash crop [11]. This facilitates the growth of the cash crop’s root system to propagate into the deeper regions of the soil profile, allowing extraction of resources from a larger soil area [12–14] and continued growth even during prolonged drought [15, 16]. Since root type, length, and density vary among plant species and soil profiles, the choice of cover crops, grown as a monoculture or as part of a mixture, is an important agricultural decision. Common cover crops belong to the families of *Brassicaceae* (mustards, crucifers, or cabbage family), *Fabaceae* (legumes), or *Poaceae* (grasses). *Poaceae* have a dense, cumulative root distribution that can be either shallow or deep. The majority of *Brassicaceae* members have a tap-rooted distribution, which allows them to penetrate deeper into the subsoil. *Brassicaceae* can also serve as green manure to increase soil fertility and organic C levels [17]. Members of the *Fabaceae* are either shallow- or deep-rooted and have a symbiotic relationship with N-fixing bacteria, producing a residual effect corresponding to 0–80 kg N ha^−1^ mineral fertilizer, which promotes soil N enrichment and N proportion in the soil together with C sequestration [18, 19], and this supports the idea of introducing them in cover crop rotations.

Root channels are hotspots of microbial C and N turnover in the soil due to the high organic matter content on their walls, which notably affects biogeochemical fluxes [20]. These channels are teeming with a vast array of microbes [21], whose populations increase compared to bulk soils alongside changes in metabolic activities [20]. The soil ecosystem as a whole plays a pivotal role in nutrient cycling, organic matter decomposition, and ecosystem health that can be explored at the functional frontier using various omics approaches. Some of the most enriched soil-associated metabolic pathways in the root zone as compared to root-associated pathways include the citric acid cycle and carbon metabolism [22], which are used by the inhabiting microbes for metabolizing organic carbon for the C cycle. Several recent studies have investigated the structures and functions of bacterial metaproteomes in soils under different conditions and environmental parameters [23–25]. The latest metaproteomic approaches have facilitated the assessment of microbial community structures and roles in metabolic pathways as a measure for quantification of biomass contributions of communities in different environments by evaluating the abundance of proteins [26, 27]. This approach can help fill in the knowledge gaps about the changes caused by the cover crops on the expression of proteins involved in the C and N cycles and how such cycles change along spatiotemporal parameters over the cash crop season. Identified proteins contributing to these cycles could be mapped to multiple bacterial taxa and unravel their roles in metabolic pathways. Additionally, we can investigate the microbial roles and changes induced by vegetation type, climate, and edaphic parameters (soil properties like pH, C fractions, moisture, and texture) [24]. Therefore, metaproteomics could be applied to understand community dynamics in biochemical pathways like N leaching and nutrient cycling and observe changes along spatiotemporal parameters of soil profile depths (topsoil and subsoil), different combinations of cash and cover crops, and their growth stages.

In this study, maize was grown as a cash crop after various perennial winter-grown cover crops. We hypothesized that reusing the cover crop root channels would allow maize to extract water and also nutrients from the deeper, larger regions of the subsoil. The objective was to structurally and functionally characterize the bacterial communities over time and depth in the maize rhizosphere, bulk soil, and root channels after cover crop growth via qPCR, 16S rRNA amplicon sequencing, and metaproteomics. The particular focus of the latter was on proteins that contribute to the C and N cycles. Additionally, improvements of microbial communities in the soil regimes with the reuse of cover crop root channels were also interpreted. Extensive field sampling was carried out to allow the identification of dominant metabolic pathways despite sample heterogeneities. Mapping the structural and functional framework of the microbiota when root channels were reused by maize will facilitate the selection of the most effective cover crops. Ultimately, this research will be helpful in the rational selection of cover crops in agricultural management practices that effectively sustain and improve soil health [28].

## Materials and methods

### Crop cultivation and sampling regimes

**Figure 1:**
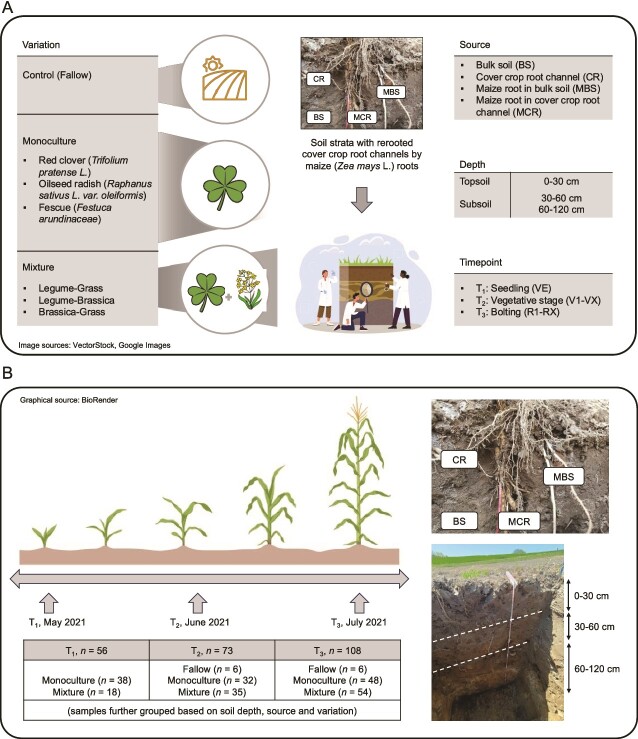
(A) A schematic overview of the experimental variables studied during this research work; (B) a schematic overview of the time points, soil profile depths, sample sources and an estimation of number of analysed samples for the different variation categories.

Crops were grown in an agricultural field at the experimental estate Hohenschulen of the Christian-Albrechts-University of Kiel (Achterwehr, Germany, 54°18′44”N, 9°59′46″E). Hohenschulen is characterized by an average annual precipitation of 806 mm and a long-term mean temperature of 8.8°C [29]. The soil type is Luvisol, which has a sandy loam texture with 17% clay in the topsoil. Plant access to subsoil resources in this soil type is hindered by a compact Bt horizon. In this study, the cash crop was maize (*Zea mays* L.), and the cover crops were shallow- and deep-rooting *Brassicaceae* (*Brassica napus* L., rapeseed, shallow-rooting; *Raphanus sativus* L. var. *oleiformis*, oilseed radish, deep-rooting); *Fabaceae* (*Trifolium repens* L., white clover, shallow-rooting; *T. pratense* L., red clover, deep-rooting); and *Poaceae* (*Lolium perenne*, perennial ryegrass, shallow-rooting; *Festuca arundinaceae*, tall fescue, deep-rooting). Seven variations were investigated: one control without cover crop; three monocultures of *Brassicaceae*, *Fabaceae*, and *Poaceae*, having combinations of deep- and shallow-rooting variants within each monoculture; and three mixtures. The mixtures were grown as a combination of shallow- and deep-rooting cover crops of *Brassicaceae*, *Fabaceae,* and *Poaceae,* complementing the niche complementary principle, which has been reported to allow polycultures to over-yield when plants compete for resources [30]. All the cover crops were sown in September 2020 in distinct randomized plots with four replicates of each variation and grew until May 2021. Plots without cover crops during the winter (bare fallow) were established as control. In May 2021, herbicide (Roundup, Bayer AG, Leverkusen, Germany) was used to kill all cover crops, and subsequently, maize was sown in the same plots and the fallow plots. Maize was growing in the field from May to September 2021 (Fig. 1A and B).

Before each sampling, the soil profile was excavated 40 cm inwards from existing profiles to obtain a fresh profile and fresh maize root system and to prevent contamination from the neighbouring soil. The samples were extracted from the profiles using a spatula and collected in plastic zip-lock bags, and the spatula was cleaned with ethanol between each sample collection to avoid contamination. Throughout the sampling, the samples were stored in coolers containing dry ice for 7–8 h (average within the same day; hours varied depending upon the duration of fieldwork) until shipment. In the laboratory, all samples were stored at −80°C until further processing. To compare the differences in the microbial community in the root channels of the cover crops with the samples from maize roots reusing the channels, we collected two types of samples from a vertical soil profile down to 120 cm in all cover crop variations: (i) material from empty cover crop root channels (CR) and (ii) material from cover crop root channels containing maize roots (MCR). The soil from empty cover crop root biopores was taken from three time points [maize germination and seedling (T_1_, vegetative emergence (VE, BBCH 0–5)], maize vegetative phase [T_2_, V1–VX (BBCH 16–20)], and shooting and bolting [T_3_, R1-RX (BBCH 35–45)]. The dead cover crop root was present in the excavated soil profile, and the soil within 2 mm of the pre-existing cover crop root was categorized as samples from the cover crop root channels [12]. The CR samples were taken from three soil layers (0–30, 30–60, and 60–120 cm) at T_1_, from 0 to 30 cm at T_2_, and from 0 to 30 and 30 to 60 cm at T_3_, to detect the cover root channel microbial communities and roles for the different cover crops. The MCR samples were collected from 0 to 30 cm at T_2_ and from 0 to 30 and 30 to 60 cm at T_3_, taking into account the maximum rooting depth of maize at each time point. Areas on the profile where fresh white maize root and dark-brown cover crop root residues completely overlap were considered MCR regions. MCR samples were soils taken within 2 mm of the roots overlap region to interpret microbial community dynamics in a potentially active region and to determine the change of the community due to the colonization of fresh maize roots in the CR.

The bulk soil (BS) samples were taken from the soil profiles from regions that were free of cover crop and maize roots. The BS samples were collected from three soil layers (0–30, 30–60, and 60–120 cm) at T_1_, from 0 to 30 cm at T_2_, and from 0 to 30 and 30 to 60 cm at T_3_ (only the samples from control profiles were collected at T_3_). Soil from the maize root zone (2 mm from the maize root) that was in the bulk soil was sampled at the sampling time points as the maize root in bulk soil samples (MBS) (Fig. 1B). The samples were categorized into the sample source groups (BS, CR, MBS, MCR) based on our visual cues. No repetition of sampling was done from the profiles of the same plot in order to avoid bias and duplicates. Due to logistical challenges, fewer samples could be taken during time point T_1_ as compared to T_2_ and T_3_, especially from fallow plots.

### DNA extraction and sequencing of 16S rRNA gene amplicons

Bacterial communities in the root zone and soil samples were analysed by sequencing of 16S rRNA gene amplicons (2 × 150 bp) on an Illumina NextSeq™ 550 (Illumina, San Diego, CA, USA). DNA was extracted from 0.25 g of soil using the DNeasy^®^ PowerSoil^®^ Pro Kit (QIAGEN GmbH, Hilden, Germany). PCR amplicons of the V3 region of the bacterial 16S rRNA gene were prepared using the forward and reverse primers 341F and 518R [31] and the NEBNext^®^ Ultra™ II Q5^®^ Master Mix (New England Biolabs GmbH, Frankfurt, Germany). Sequencing libraries were prepared from 100 ng of DNA according to the Illumina protocol. Dual index adapters for the sequencing were attached using the NEBNext Multiplex Oligos for Illumina. The final concentration of the libraries was 2 nM after pooling. We sequenced triplicates of samples from each soil depth and soil/root zone source per cover crop variation plot for all three sampling time points (*n* = 237).

The sequencing data were analysed using QIIME2 v2023.2 [32]. First, the raw sequence reads were demultiplexed and quality-filtered (*q*-score ≥ 20) using the q2-demux plugin, followed by denoising with DADA2 [33] (via q2-dada2). Both the 16S forward and reverse sequences were trimmed at 130 bp. All amplicon sequence variants (ASVs) were aligned with mafft [34] (via q2-alignment), and then, maximum-likelihood trees were constructed using FastTree2 [35] (via q2-phylogeny). We chose ASV-based methods over operational taxonomic unit (OTU) approaches to limit the effect of spurious taxa on diversity indices [36]. Taxonomic assignment of ASVs was carried out using the q2-feature-classifier [37] and the classify-sklearn Naïve Bayes taxonomy classifier against the SILVA v138.1 “99 % OTU reference sequences” [38].

### Quantitative PCR

The copy number of the 16S rRNA gene per gram of soil was quantified by SYBR^®^ Green-based qPCR using a 7500 Fast Real-Time PCR System (Applied Biosystems™, Thermo Fisher Scientific, Waltham, MA, USA). Aliquots of the same DNA extract utilized in amplicon sequencing were used for qPCR. Dilutions of template DNA were used to compensate for the effect of PCR inhibitors in the samples. Each sample was analysed in triplicate. A PCR amplicon of the *Escherichia coli* V3 region was used as the standard. Each 20 μl reaction contained 1 μl of template DNA, the forward and reverse primers 341F and 518R without adapter nucleotides [39], and Luna^®^ Universal qPCR Master Mix (NEB). Reaction conditions were an initial denaturation for 1 min at 95°C, followed by 40 cycles of denaturation at 95°C for 15 s and extension at 60°C for 30 s. The melting curve was recorded in the temperature range of 60°C–95°C. The 16S rRNA gene copy numbers per gram of soil were determined in comparison against the standard essentially as before [40]. The average efficiency value was 100.8 ± 3.2%. For quantitative microbiome profiling [41], the absolute copy numbers per gram of sample for each bacterial phylum were calculated by multiplying the qPCR values by the relative abundance values in % obtained from the 16S rRNA gene sequencing analyses, as shown by the equation:


$$ {n}_{cn}=a\times{b}_{cn} $$

where *n_cn_* is the absolute copy number of each phylum per gram of soil, *a* is the relative abundance of each phylum in percentage, and *b_cn_* is the absolute copy number per gram of soil.

### Metaproteomics analysis

At each time point, samples were collected separately from three plots for each cover crop variation at the analysed soil depths and sample sources and used for proteomic analyses following a previously described protocol [42]. Approximately 4 g of soil (from the same set of samples used for 16S rRNA sequencing) was used for protein extraction using the Sodium dodecyl sulfate (SDS) buffered-phenol extraction method as previously described [43]. The protein extract was purified using 1D SDS-PAGE, and then the extract was further digested with trypsin. A nano-High-performance liquid chromatography (HPLC) system (UltiMate™ 3000 RSLCnano system, Thermo Fisher Scientific, Waltham, MA, USA) was used to separate the cleaved peptides. The system was connected to a Q-Exactive HF Orbitrap Liquid chromatography-mass spectrometry (LC-MS/MS) system (Thermo Fisher Scientific) equipped with a nanoelectrospray ion source, Triversa NanoMate^®^ (Advion, Ithaca, NY, USA). We linked the MS data to an in-house-generated proteome database containing all the defined proteomes in UniProt for the bacteria identified in the samples by 16S rRNA amplicon sequencing. The database search was performed with Proteome Discoverer™ (v2.5.0.8, Thermo Fisher Scientific) using the SEQUEST-HT algorithm. The precursor mass tolerance of the MS was set to 10 ppm, and the fragment mass tolerance of the MS/MS was 0.02 Da. Carbamidomethylation of cysteine was considered fixed, and oxidation of methionine was set as a dynamic modification. Enzyme specificity was set to trypsin with up to two missed cleavages allowed using 10 ppm peptide ions and 0.02 Da MS/MS tolerances. Only rank-one peptides with a Percolator-estimated false discovery rate <1% were accepted as identified. The GhostKoala [44], Kyoto Encyclopedia of Genes and Genomes (KEGG) [44], Clusters of Orthologous Groups of protein (COG) [45], and carbohydrate-active enzymes (CAZy) [46] databases were used for protein functional annotation. Pathways with a minimum of two proteins and a minimum coverage of 5% were selected for downstream processing. The CAZy enzymes were identified using peptide analyses of Unipept Desktop (v2.0.0, Ghent University) [47]. The identified CAZy enzymes and their preferred substrates provide information regarding rhizo-deposits in the soil profile along the cover crop root channels. We analysed the measured label-free quantification (LFQ) intensities of the identified proteins and determined the functional metabolic pathways to detect changes along the soil depths, maize growth stages, and cover crop variations. We used the bacterial taxa identified via 16S rRNA sequencing for the reference database construction, as previously implemented [48]. During the construction of the reference database from UniProt for mapping the identified proteins to respective taxonomic communities, we made sure of minimum redundancy with maximum relevancy to negate repetitive identification of already-measured proteins, which would skew the observations. In the analysis, proteins that were unambiguously identifiable by unique shared peptides were grouped together as “protein groups” and used for quantifications. Using the ENTREZ key, the National Center for Biotechnology Information (NCBI) database [49, 50] was used for obtaining the taxonomic information for each identified protein using unique protein identifiers called KEGG Orthology (KO) identifiers. Each functional pathway had unique KEGG and COG identifiers, which we linked to proteins and protein groups to connect respective functional pathways, followed by the NCBI-linked KO identifiers to integrate taxonomic communities with functional pathways. The LFQ values were highly variable for the identified protein groups. So, the measures were normalized by log2-transformation using the *log* function of base R (v4.3.1) [51] prior to any graphical representations or statistical significance tests. After categorizing proteins into different functional pathways, we focused specifically on the C and N cycles to understand the changes along the spatiotemporal parameters with the introduction of reusage of cover crop root channels and find suitable cover crop choices.

### Quantifying microbial biomass carbon and nitrogen

Soil microbial biomass C and N were determined using the chloroform fumigation extraction method [52, 53]. In brief, 7.5 g of soil was fumigated with chloroform for 24 h and then extracted with 30 ml of 0.05 M K_2_SO_4_ on a shaker for 1 h. A nonfumigated soil sample was treated in parallel with the observations of the fallow samples. C and N were measured with the N/C 2100 TOC/N analyser (Analytik Jena, Jena, Germany). microbial biomass of C (MBC) was calculated as the difference between extracted C from fumigated and nonfumigated soil with a conversion factor (*k_C_*) of 0.45 [54]. microbial biomass of N (MBN) was calculated as the difference between extracted N from fumigated and nonfumigated soil with a conversion factor (*k_N_*) of 0.54 [54, 55]. The quantification of MBC and MBN were presented as μg g^−1^ dry soil. For each of the treatment and fallow samples, a total of four replicates were measured, and their means were calculated.

### Statistical data analysis

We used R (v4.3.1) [51] to perform all statistical analyses of the sequencing and the metaproteomics data. All measures of significance were calculated using multivariate analysis of variance (ANOVA) and linear mixed models, followed by Tukey’s range *post hoc* test (TukeyHSD) with package *stats* (v3.6.2) and *rstatix* (v0.7.2) [56]. In the 16S rRNA sequencing analysis, the ASV abundance tables were filtered with total-frequency-based filtering based on 95% sequence identity (via q2-feature-table summarize) and rarefied at 5000 sequences to ensure equal sampling depth and sorting in the maximum number of samples for diversity analyses. Alpha and beta diversity metrics were calculated using the packages *phyloseq* [57] and *microbiome* [58] from R (v4.3.1) [51]. The number of “observed ASVs” measured for each cover crop variation at different sampling time points and depths was used for calculating alpha diversity richness. Pielou’s evenness is the most widely used diversity evenness index in the ecological literature [59]. For beta diversity, we used weighted UniFrac distance units [60] and visualized differences via Principal Coordinate Analysis (PCoA) using the *vegan* package (v2.6-4) [61]. Using the linear mixed model, we evaluated the significantly different cover crop variations using time points and sampling depths as random effects and cover crop variations as the fixed effect. Additionally, Tukey’s honestly significant difference (HSD) was chosen for multivariate significance tests with parameters of source, variation, depth, time points, and bacterial phyla. A four-way permutational multivariate ANOVA (PERMANOVA) among the parameters of source, variation, depth, and time points was used to quantify the significance among the parameters based on UniFrac distance using *adonis2* of the *vegan* package (v2.6-4) [61]. For bacterial abundances, the significance between the parameters was represented using the compact letter display (CLD) [62] with the help of the *multcompLetters* package [63]. For metaproteomics, the significantly different cover crop variations or proteins of different metabolic pathways or bacterial phyla were calculated using multivariate ANOVA, using source, variation, depth, time points, and bacterial phyla as fixed factors. Upon determination, they were represented by significant stars based on the adjusted *P*-values (^*^P < .05, ^**^P < .01, ^***^P < .001, ^****^P < .0001). All figures were generated in RStudio using the packages *ggplot2* (v3.4.2) [64], *cowplot* (v1.1.1) [65], and *phyloseq* (v1.44.0) [57]. Other integrated packages used for statistical analyses and figure generation were *tidyverse* (v2.0.0), *dplyr* (v1.1.3), and *splitstackshape* (v1.4.8) [66].

We linked protein intensities and their source taxa from metaproteomics with the absolute abundance from qPCR measurements for the respective bacterial taxa. Taxonomic information from both 16S rRNA sequencing and metaproteomics allowed crosslinking of the communities and identifying the ones overproducing proteins of various pathways of the C and N cycles. The relative expression levels of proteins involved in the C and N cycles were estimated as follows. First, the proteome LFQ intensities and the 16S rRNA gene copy numbers of each phylum per gram of soil were individually normalized by *min-max* normalization in the range of zero to one using the rescale function from the *scales* (v1.2.1) package [67] of R (v4.3.1) [51]. To identify overproduced enzymes and their host phyla, the ratios of the rescaled LFQ intensities to the rescaled 16S rRNA gene copies per gram of soil were quantified. The ratio threshold was 1 if the rescaled protein abundance was equal to the rescaled bacterial abundance. When the ratio was >1, the enzymes were classified as overproduced by the particular phylum in the cover crop variation from which the sample was collected.

## Results

### Bacterial community structure in the root zone and bulk soil

Bacterial communities in the bulk soil and the soil around maize growing after different cover crop variants were characterized by 16S rRNA amplicon sequencing and quantified by qPCR. A total of 42.6 million high-quality reads were generated from 237 samples, yielding 22 309 unique ASVs that were assigned to 41 bacterial phyla (Supplementary Fig. S1). The community comparisons based on alpha and beta diversity index calculations are first described, and then, the abundances of total bacterial 16S rRNA gene copy numbers and predominant phyla over time, space, and cover crop variation are reported.

**Figure 2:**
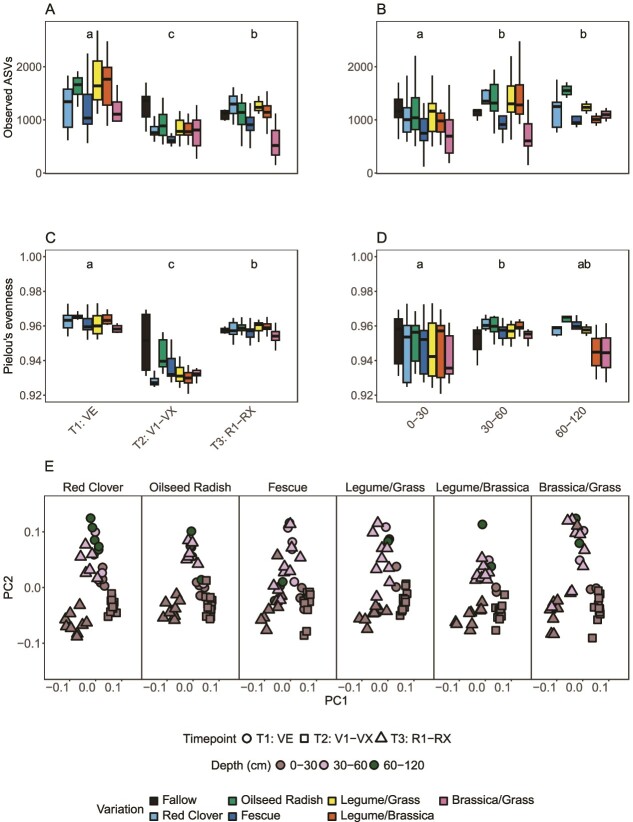
(A, B) Observed ASVs reflecting community richness for the growth phases of maize and depths of the soil horizon from all soil sample sources combined (BS, CR, MBS, and MCR) over the variations of cover crops. The time points are T_1_: VE (seedling), T_2_: V1–VX (the vegetative phase), and T_3_: R1–RX (bolting). The depths from which the soil samples were collected are topsoil (0–30 cm) and subsoil (30–60, 60–120 cm). Pairwise correlation between the variations is shown using a compact letter display representation for the significant differences between the variations calculated using Tukey’s range test (TukeyHSD); (C, D) Pielou’s evenness represents species evenness of the maize growth stages and depths along the cover crop variations; (E) bacterial community beta-diversity visualized using PCoA ordination based on weighted UniFrac distances along maize growth stages and depths for each cover crop variation; for T_1_, *n* = 56; for T_2_, *n* = 73; for T_3_, *n* = 108; for depth (topsoil and subsoil), *n* = 237; CLD values: time points: T_1_—“a,” T_2_—“c,” T_3_—“b”; depths: 0–30 cm—“A,” 30–60 cm—“b,” 60–120 cm—(“b” for richness, “ab” for evenness); (Supplementary Table S1i–l provides the data for pairwise tests for significance for the 16S rRNA ASVs for phylotype richness and evenness along the parameters of sampling time points and depths, followed by CLD representations).

The differences in alpha and beta diversity were largest between samples from different time points and depths (Fig. 2). However, there were also statistically significant differences between variants. Specifically for the different sampling time points based on maize growth stages, the number of ASVs as a measure of community richness was significantly the highest during VE (T_1_, BBCH 0–5) with an average of 1470, then decreased to 847 in V1–VX (T_2_, BBCH 16–20), and increased again during R1–RX (T_3_, BBCH 35–45) to an average of 1080 (^*^P = .04, Fig. 2A, Supplementary Fig. S2A, Supplementary Tables S1A and S2). Community richness was significantly higher in the two mixtures with *Fabaceae* at T_1_ (1772 and 1678 ASVs) due to the highest abundance of rare ASVs and lowest in the *Poaceae* monoculture of fescue at T_2_ (616 ASVs) and in the mixture *Brassicaceae*/*Poaceae* at T_3_ (632 ASVs).

For the different sources of soil samples, richness in the samples CR, MBS, and MCR was significantly higher in the community profiles of *Fabaceae* and *Brassicaceae* monocultures (red clover–1147 ASVs, oilseed radish–1123 ASVs) and mixtures (*Fabaceae*/*Brassicaceae*, 1142 ASVs) as compared to *Poaceae* monoculture (fescue, 832 ASVs) and mixture with *Brassicaceae* (*Brassicaceae*/*Poaceae*, 693 ASVs) (Supplementary Table S1c). Along the soil sampling depth profile, alpha diversity increased slightly from an average of 1008 ASVs in the topsoil (0–30 cm) to an average of 1204 in the two subsoil sampling depths (30–60 and 60–120 cm) (^*^P = .04, Fig. 2B, Supplementary Fig. S2B, Supplementary Tables S1B and S2). Community evenness values showed a similar picture over time, with almost the same average Pielou’s evenness for T_1_ and T_3_ (0.960 _avg._ and 0.956 _avg._), while both were higher than T_2_ across all cover crop variations (0.936 _avg._, ^**^P = .001) (Fig. 2C, Supplementary Tables S1D and S2). Community evenness, however, was rather similar along the depth of the horizon in the topsoil (0.948 _avg._) and the subsoil across all variations (0.955 _avg._) (Fig. 2D, Supplementary Table S1E). Additionally, the effect of spatiotemporal parameters (soil sample depths and the maize growth stages) on cover crop variations showed the mixture of *Brassicaceae* with *Poaceae* as significantly different from other cover crop variations both in the topsoil and subsoil during V1–VX and R1–RX (^*^P = .023, Supplementary Table S1h).

Beta diversity was visualized by PCoA of weighted UniFrac distances, with results for time points and depths shown in Fig. 2E. PERMANOVA and ANOVA statistical tests show that communities at T_1_, T_2,_ and T_3_ were statistically significant from each other, as well as the topsoil communities against those from subsoil samples (^****^P = 1.71 × 10^−5^, Supplementary Tables S2 and S3a,b, and d). Notably, communities in topsoil at T_2_ were statistically significant than those from T_1_, T_3_, and subsoil (^***^P = 6.56 × 10^−3^, Supplementary Table S3e). The trend was similar in all the cover crop variations as well as in fallow. Regarding sample types, those collected from BS were also statistically significant from MCR (^*^P = .04, Supplementary Table S3c), shown as separate clusters of bacterial communities between the bulk soil and the root zone (Supplementary Fig. S3).

**Figure 3:**
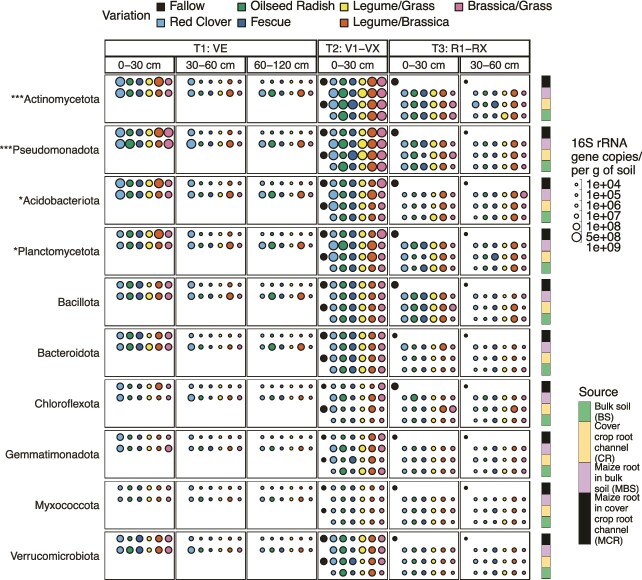
The absolute normalized abundance of 16S rRNA gene copies per gram of soil for the different variations was categorized based on the time points of the maize growth (T_1_: VE [seedling], T_2_: V1–VX [the vegetative phase], and T_3_: R1-RX [bolting]); the depth of the collected samples (0–30, 30–60, 60–120 cm); and the different sample sources (BS, CR, MBS, MCR). The number of samples used for the temporal calculation was *n* = 56 for T_1_, *n* = 73 for T_2_, and *n* = 108 for T_3_. The asterisks with the names of phyla represent significant differences among the different variations considering the 16S rRNA gene copies per gram of soil for the specific variations and the linked phyla; ^*^P < .05, ^**^P < .01, ^***^P < .001, ^****^P < .0001; (Supplementary Table S3d provides the data for pairwise tests for significance for the 16S rRNA gene copies along the parameters of variations and the bacterial phyla).

The average number of total 16S rRNA gene copies per gram of soil increased slightly from T_1_ to T_2_ and then decreased by approximately ${}^{1}\!\left/ {}_{3}\right.$ order of magnitude when maize reached T_3_ (2.4 × 10^9^_T1 (topsoil)_, 2.7 × 10^9^_T2 (topsoil)_, and 1.0 × 10^9^_T3 (topsoil)_ copies/g; 4.1 × 10^8^_T1 (subsoil)_, and 3.6 × 10^8^_T3 (subsoil)_ copies/g; Fig. 3, Supplementary Table S4A). The average copy numbers in the topsoil were almost an order of magnitude higher than in the subsoil (2.1 × 10^9^_0–30 cm_, 3.9 × 10^8^_30–60 cm_, and 3.3 × 10^8^_60–120 cm_ copies/g; ^****^P = 2.0 × 10^−6^, Fig. 3, Supplementary Tables S3B and S4A). The mixture of *Fabaceae*/*Brassicaceae* had the highest 16S rRNA gene copies in the majority of sample types [3.2 × 10^9^_BS (topsoil)_, 2.3 × 10^9^_MBS (topsoil)_, and 1.9 × 10^9^_MCR (topsoil)_ copies/g; 1.4 × 10^9^_CR (subsoil)_, 1.5 × 10^9^_MBS (subsoil)_, and 1.0 × 10^9^_MCR (subsoil)_ copies/g; Fig. 3, Supplementary Table S4A]. The *Fabaceae* monoculture red clover had the highest copy numbers in the CR of the topsoil and in the BS from the subsoil [2.6 × 10^9^_CR (topsoil)_, 1.0 × 10^9^_BS (subsoil)_ copies/g; Fig. 3, Supplementary Table S4A].

The taxonomic composition of the communities is reported at the phylum level in order to have the same level as for the metaproteomics results (see below), for which a greater taxonomic resolution for complex systems is often not possible since peptide sequences are too short/not divergent enough. *Acidobacteriota*, *Actinomycetota*, *Bacillota*, *Bacteroidota*, *Chloroflexota*, *Planctomycetota,* and *Pseudomonadota* were the predominant bacterial phyla (Fig. 3, statistically significant phyla tabulated in Supplementary Table S3d), all of which are frequently detected as abundant members of the rhizosphere and the microbiome associated with maize and other plants [68–71]. All the above-mentioned bacterial phyla increased in all communities with the introduction of cover crop root channel reusage against fallow. *Acidobacteriota* and *Planctomycetota* increased from VE (T_1_) to V1–VX (T_2_) of the maize and then decreased towards R1–RX (T_3_) (Fig. 3, Supplementary Fig. S4). Furthermore, *Bacillota* and *Chloroflexota* increased relatively from V1–VX to R1–RX (Supplementary Fig. S4).

### Metaproteomic insight into microbial metabolic pathways

A total of 36 077 proteins belonging to 3677 different protein groups were identified, with an average of 626 protein groups per variant across all sample types and an average of 1066 in the samples from root zones (Supplementary Tables S5a and b). The latter number of unique bacterial proteins is higher than in previous reports on metaproteomics for the maize rhizosphere (548–806 proteins) [72, 73], showing comparable high metaproteome coverage in this study. The total number of protein groups was higher during VE (T_1_, 1042 _avg._) and V1–VX (T_2,_ 788 _avg._) than in the R1–RX stage (T_3,_ 363 _avg._), resembling the absolute abundance counts of the 16S rRNA gene. Similarly, protein group numbers decreased from an average of 424 in the topsoil (0–30 cm, T_3_) to an average of 291 in the subsoil (30–60 and 60–120 cm, T_3_). The two subsoil regimes of 30–60 and 60–120 cm did not display statistically significant changes in the total microbial biomass of C and N in the maize root zone (*P* > .05, Supplementary Tables S6a and b), which also signifies lesser fluctuations. Henceforth, we categorized them together as subsoil rather than considering them individually. The number of identified protein groups was higher in samples from reused root channels in the topsoil and subsoil, both for cover crop monocultures and mixtures, compared to samples from fallow plots. In the subsoil, we observed an increase in the number of protein groups following the reusage of root channels by the cover crops. With the growing maize reaching the subsoil after T_2_, the reused root channels had an average of 389 protein groups in the subsoil at T_3_ as compared to 88 for fallow. This implied an increased active metabolism in the subsoil once the reusage of root channels came into effect. The metadata of all identified enzymes linked to the C and N cycles is given in Supplementary Tables S7a and b and abundance heatmaps for the identified enzymes corresponding to the distinct bacterial phyla in the different variations and the growth stages of maize (Supplementary Figs S5 and S6).

**Figure 4:**
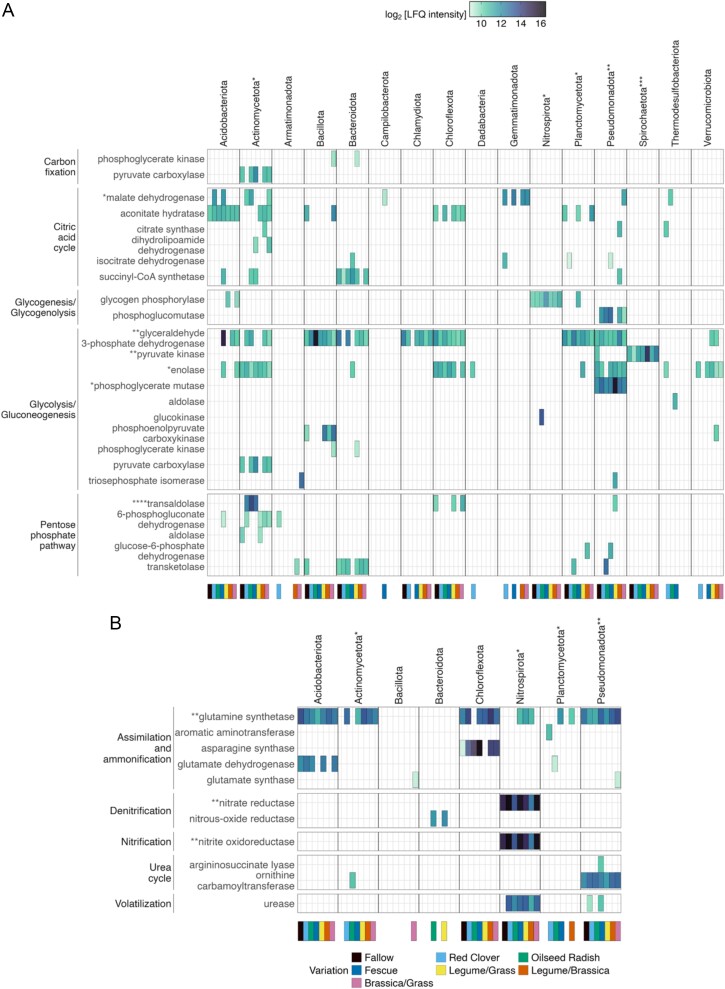
(A, B) Alterations of enzymes in the C and the N cycles in the different cover crop reuse variations. A phylum-specific expression of enzymes in each of the variations illustrates the contribution of different bacterial phyla to the multiple steps of the C and N cycles. The asterisks with the names of phyla and enzymes represent significant differences among the variations considering the LFQ intensities of enzymes and the linked phyla. Log_2_-transformed values of the LFQ of the protein intensities were used for the heatmap and statistical analyses; ^*^P < .05, ^**^P < .01, ^***^P < .001, ^****^P < .0001 (*n* = 232) (Supplementary Table S9a provides the data for pairwise tests for significance for the steps of the C and N cycles along the parameters of variations and the bacterial phyla).

**Figure 5:**
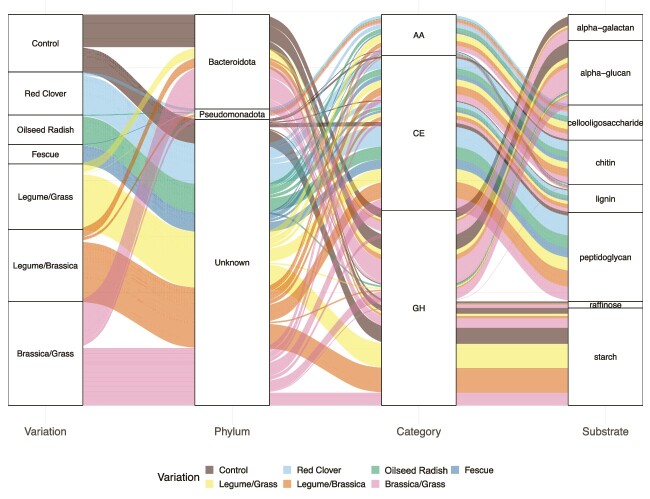
An alluvial plot representing the different categories of CAZymes from the distinct bacterial phyla and the substrates they act upon in the distinct variations; AA, auxiliary-active enzymes; CE, carbohydrate esterases; GH, glycoside hydrolases.

Most of the identified proteins were assigned to carbohydrate, energy, and amino acid metabolism, with *Pseudomonadota* being the major contributor (Fig. 4A and b). Across the different cover crop variations, there were significant differences in the expression of enzymes involved in the citric acid cycle, glycolysis, and pentose phosphate pathways (Fig. 4a and b, Supplementary Figs S7 and S8, Supplementary Tables S8A and B and S9B [with significant pairwise comparisons only]). The monocultures and mixtures of *Fabaceae* and *Brassicaceae* had significantly higher expression of enzymes involved in the C cycle steps than *Poaceae* (^**^P = .0019, Supplementary Tables S8A and B and S9B). Additionally, glycoside hydrolases (GHs) were the most abundant CAZy enzymes [46] (Fig. 5). The most highly abundant of the identified GHs act on galactans and glycans as substrates (Supplementary Table S10). The phylum *Bacteroidota* was the largest contributor to CAZy enzymes. Additional identified CAZy enzymes were carbohydrate esterases catalysing reactions with lignin, chitin, and peptidoglycans as substrates and auxiliary active enzymes metabolizing cellooligosaccharides. In line with total protein abundances in our metaproteomic dataset, the quantity of CAZy enzymes was higher in reused root channels of cover crop mixtures as compared to fallow.

**Figure 6:**
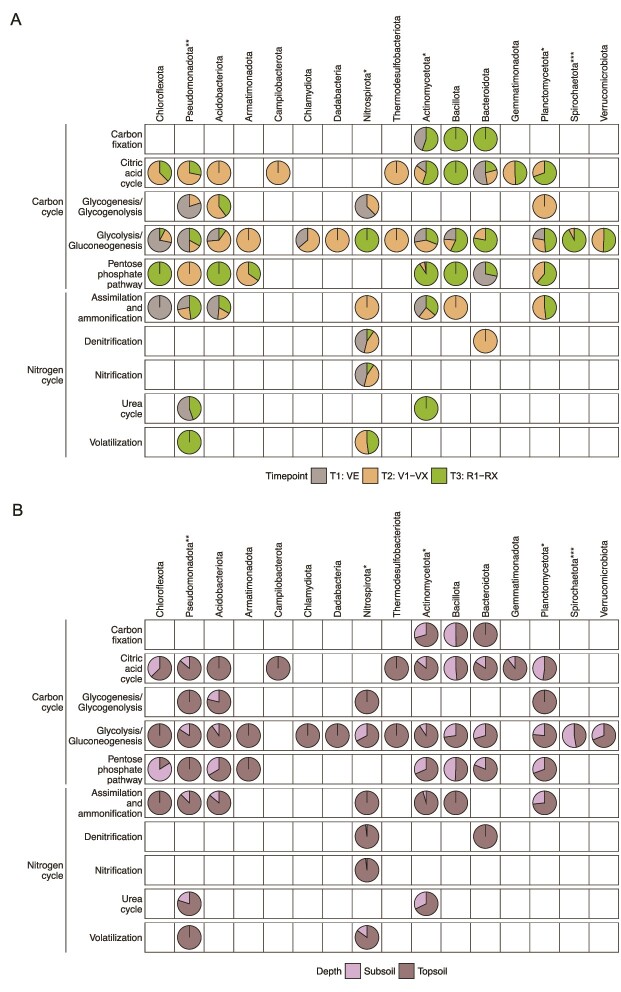
(A) A trend for the expression of the different steps of the C and N cycles along the three distinct time points of the maize growth phase. The pie-chart measurements were calculated as a percentage of the overall expression of enzymes in all the steps involved in the C and N cycles, for each bacterial phylum and maize growth stages. The empty spaces indicate no identification of enzymes involved in the steps from the specific phyla, as observed in the metaproteomic analysis. The asterisks with the names of phyla represent their significant differences among the variations; (B) A similar trend but along the depth of soil profiles. The pie-chart measurements were calculated as a percentage of the overall expression of enzymes in all the steps involved in the C and N cycles, for each bacterial phylum and rhizosphere horizon depths. The asterisks with the names of phyla represent their significant differences among the different variations; ^*^P < .05, ^**^P < .01, ^***^P < .001 (Supplementary Table S9a-d provides the data for pairwise tests for significance for the steps of the C and the N cycles along the parameters of variations, soil profile depths, maize growth stages, and the bacterial phyla).

Furthermore, statistical tests showed that the abundances of proteins involved in nitrification and dissimilatory nitrate reduction were significantly higher in the two mixtures with red clover than in the other variations (^**^P = .0012, Supplementary Table S8b). Elevated nitrite oxidoreductase (NXR) and dissimilatory nitrate reductase (NAR) abundance in the monocultures and mixtures of *Fabaceae* and *Poaceae* during V1–VX indicates enhanced nitrification of NO_2_^−^ to NO_3_^−^ as well as denitrification in the soil, apparently driven by a larger *Nitrospirota* community (Fig. 6A). Furthermore, proteins involved in secondary metabolite synthesis and nucleotide transportation were more abundant in the monocultures and mixtures with *Fabaceae* and *Brassicaceae* (Supplementary Tables S9b and S11).

### Bacterial phyla overproduce carbon-cycle enzymes

**Figure 7:**
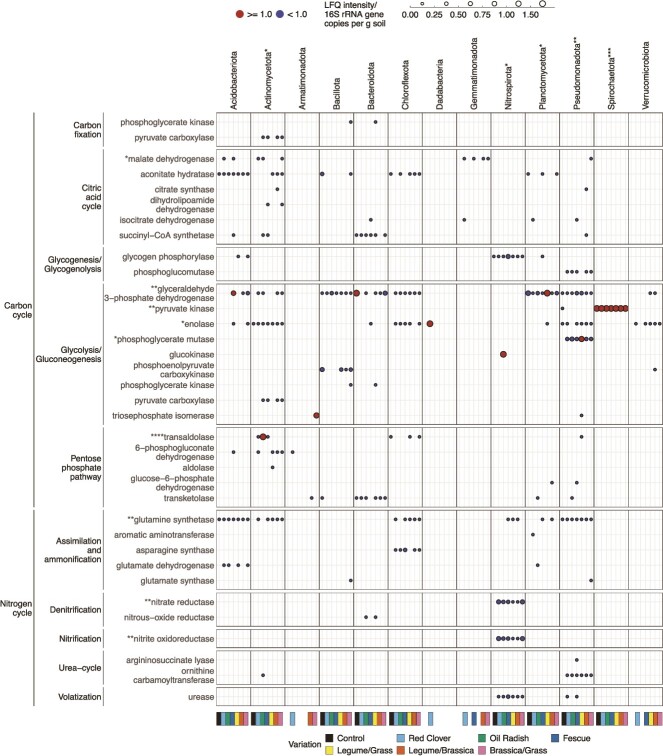
Enzymes in the different steps of the C and N cycles were identified by dividing the LFQ intensity from metaproteomics with the relative 16S rRNA gene copies per gram of soil, after performing *min-max* normalization on both datasets. The size of the circles represents the magnitudes of the ratio. The asterisks with the names of phyla and enzymes represent significant differences among the variations considering the LFQ intensities of enzymes and the linked phyla; ^*^P < .05, ^**^P < .01, ^***^P < .001, ^****^P < .0001 (Supplementary Table S9a provides the data for pairwise tests for significance for the steps of the C and N cycles along the parameters of variations and the bacterial phyla).

To identify overproduced proteins involved in the C and N cycles, the ratios of normalized LFQ intensities vs. normalized phylum abundances were calculated. In the C cycle, enzymes involved in glycolysis and the pentose phosphate pathway were overproduced by *Acidobacteriota*, *Actinomycetota*, *Armatimonadota*, *Bacteroidota*, *Dadabacteria, Nitrospirota*, *Planctomycetota*, *Pseudomonadota*, and *Spirochaetota* (Fig. 7, Supplementary Table S12). Specifically, enolase, glucokinase, glyceraldehyde 3-phosphate dehydrogenase (GAPDH), phosphoglycerate mutase, pyruvate kinase, and transaldolase were identified as overproduced enzymes. No N cycle enzymes were found to be overexpressed by the identified bacterial communities.

Upregulation of C-cycle enzymes was found in most of the variations of monocultures and mixtures but with some differences among the variations. The activities of glycolysis and the pentose phosphate pathway differed significantly among the different variations (^***^P < .001, Supplementary Table S9b), with the cover crop mixture variations of cover crops showing higher expression of enzymes than the monocultures, implying a comparative increase in the expression of bacterial enzymes involved in the turnover of hexoses and pentoses more than other C-cycle enzymes after cover cropping. The glycolytic enzymes were more abundant in the respective *Brassicaceae* and *Poaceae* monocultures of oilseed radish and fescue and the mixtures of *Brassicaceae*/*Poaceae* and *Fabaceae*/*Poaceae*, whereas transaldolase from the pentose phosphate pathway was overproduced in the *Brassicaceae* monocultures. Furthermore, the overproduced enzymes were from different bacterial phyla in the different variations. GAPDH was overproduced by *Acidobacteriota* in *Poaceae* monocultures, whereas it was contributed by *Planctomycetota* in the mixture of *Fabaceae*/*Poaceae*.

## Discussion

### Temporal dynamics in bacterial communities in the maize root zone

The complexity of soil as a habitat, including its substantial small-scale heterogeneities, presents an experimental challenge to gain reliable and general insights into the *in situ* microbial communities. Here, we analysed 237 samples from one soil type, Luvisol, with maize grown after cover crops using qPCR, amplicon sequencing, and metaproteomics. To demonstrate that the number of samples taken allowed a comprehensive and reliable analysis of microbial community features, we first discuss the abundance and distribution of different bacteria and their metabolic traits based on the microbial–ecological recognition that root exudates are the plausible primary bacterial source of carbon and metabolic energy in the root zone [74].

A significant increase in 16S rRNA gene copies in root zone samples of maize reusing pre-existing cover crop root channels was observed from VE (T_1_) to V1–VX of maize (T_2_), with a subsequent decrease in R1–RX (T_3_). With reference to previously published research studies, it appears that the pattern of our observations is consistent with the change in the synthesis rate of photosynthates and a plausible change in the release of root exudates along the growth phases of maize [75, 76]. The number and biomass of bacteria and their activity in the root zone increase as rhizodeposition increases [74, 77, 78]. The microbial community had slightly fewer phylotypes during V1–VX, some of which had a higher relative abundance than during VE and R1–RX. Similar temporal trends in alpha diversity have been previously reported for soil and root zone microbiomes associated with maize [79–81]. The concomitant decrease in alpha diversity, especially Pielou’s evenness, at T_2_ indicates an increased availability of labile organic carbon in the root zone, as reported previously in soil carbon cycling [82]. This provides a competitive advantage for some bacterial phyla, e.g. members of the *Acidobacteriota*, *Planctomycetota*, and *Pseudomonadota*. Previously, *Acidobacteriota* was observed to increase from VE (T_1_) to V1–VX (T_2_) of the maize and then decrease towards R1–RX (T_3_) due to the decrease in plant-derived organic matter after V1–VX [83]. At T_3_, members of the *Bacillota* and *Chloroflexota* increased in prevalence. Members of these two phyla have been reported to increase in O_2_-deficient soil [84] and their preference for low-nutrient conditions [85, 86]. The relative increase in *Bacillota* and *Chloroflexota* abundances could be due to their capability to degrade complex organic carbon and their tolerance to low fluxes of metabolic energy [87, 88]. The observed temporal shift in beta diversity of the bacterial communities accentuated that the composition and fluxes of root exudates could have changed along the growth stages.

The metaproteomics results further defined the temporal difference in organic C fluxes, as there was a noticeable increase from T_1_ to T_2_ in enzymes involved in metabolizing hexoses and pentoses, in the citric acid cycle, and in the N cycle (Fig. 6A). The phyla *Acidobacteriota*, *Actinomycetota*, *Nitrospirota*, and *Pseudomonadota* contributed prominently to the total abundance of these enzymes. Furthermore, the detection of CAZy enzymes via metaproteomics provided evidence for the metabolization of more complex root exudates, such as galactans and glycans, into simpler organic molecules. Out of the 41 identified bacterial phyla, the abundant phyla *Actinomycetota*, *Bacillota*, *Bacteroidota*, *Planctomycetota,* and *Pseudomonadota* were found to be producers of CAZymes, with *Bacteroidota* being the most prolific source of these enzymes (Fig. 5). This was also observed previously using metagenomics in other soils [89, 90], where members of the *Bacteroidota* are known for polysaccharide-degrading properties, especially hemicellulose, and this matches our metaproteomic observations for CAZymes. A recent study reported that increased rhizodeposition could result in stimulated denitrification [91]. In our case, we observed elevated levels of dissimilatory NAR during V1–VX that could result in enhanced denitrification and ultimately facilitate the prevention of nitrate leaching.

### Topsoil and subsoil differentiation of bacterial abundances and functions

The topsoil had significantly higher absolute abundances of bacteria and enzymes from the C and N cycles compared to the subsoil, as shown by our DNA-based analyses and metaproteomics. The denser root network in the topsoil leads to more rhizodeposition of exudates and secretion and leakage of sugars, amino acids, and organic acids [92]. A positive correlation between root exudation and surface root area indicates higher root exudation in the topsoil with a denser root network with a higher surface root area [92]. Several previous studies showed that the depth of the soil horizon impacts environmental gradients, which affect the bacterial community. Hao *et al.* [93] reported a decrease in community diversity and richness with increasing depth in agricultural fields with maize and soybean (*Glycine max*), and Frey *et al.* [94] showed that bacterial abundance decreased with depth in Cambisol, Leptosol, and Regosol soils at forest sites, which correlated with decreasing C and N stocks with depth [95, 96]. Here, the spatial separation between the soil layers was further reflected by shifts in beta diversity in all studied cover crop variations. A marginal increase in community richness was also observed in the subsoil as compared to the topsoil at both bulk soil and root zone (CR, MBS, MCR) (Supplementary Fig. S2B, Supplementary Tables S1B and C). While contrasting to several previous reports [84, 97, 98], this could have been caused by a relative increase in low-abundant phylotypes in the deeper sections of the soil horizon. The increase in ASVs in the subsoil was largely due to an increase in the relative abundance of more diverse *Bacillota*, *Chloroflexota,* and the lesser abundant phyla *Methylomirabilota* and *Zixibacteria* at the expense of a decrease in dominant phyla *Actinomycetota*, *Planctomycetota*, and *Pseudomonadota*. These observations were complemented by the increase in 16S rRNA gene copy numbers from BS to the root zone soil sources (CR, MBS, and MCR) in the subsoil. *Bacillota*, *Chloroflexota*, and also *Spirochaetota* play important roles in metabolism in the subsoil [84, 85, 98–101]. Many of these bacteria can survive in low-nutrient environments and have the capability to process complex organic carbon [87, 88] (Fig. 6B, Supplementary Fig. S9). The comparative increase in their relative abundances in the subsoil compared to the topsoil has been observed across different drainage conditions with grasses and shrubs grown in a mountainous environment [84]. We hypothesize that due to the easier degradation of readily accessible simpler organic carbon among the root exudates such as sugars like glucose and pentose by *Pseudomonadota* [102] and xylose by *Bacteroidota* [103], the remaining complex organic carbon comparatively increases in proportion, especially in the subsoil after root channel reuse. This increase in complex organic carbon likely caused a relative increase in members of the *Bacillota* and *Chloroflexota* in the lower soil horizon. Similarly, the relative increase of *Spirochaetota* in the subsoil may be explained by their ability to degrade cellulose and low-molecular-weight organic matter under anaerobic conditions [104, 105].

We speculate that the higher levels of C and N microbial biomass in the topsoil compared to the subsoil (Supplementary Tables S6a and b) were likely due to the greater fluxes of maize rhizodeposition in the topsoil. Likewise, an increase in C and N in root channels reused by maize roots in the subsoil was the result of increased rhizodeposition and therefore increased bacterial abundances. An increasing protein number of groups in the subsoil after the reuse of cover crop root channels by the growing maize is an indicator of enhanced metabolic dynamics in the deeper regions of the root zone. The established root channel architectures of cover crops facilitate deeper propagation of maize roots into the subsoil, as observed via increasing bacterial abundance and protein groups in the subsoil. This could possibly allow the extraction of untapped nutrient reserves and also have rhizo-deposits utilized by microorganisms such as *Bacillota*, *Chloroflexota*, and *Spirochaetota*, which we have seen to prosper in the subsoil conditions. The presumably higher rhizodeposition also allows prolonged C and N depositions during the crop growth phase [74, 106–109], i.e. the nutrients in organic residues will be released over a longer period, providing sustained nourishment for the crops, especially in the root channel–maize root interaction zone. Therefore, establishing inroads into the deeper subsoil benefits both the subsoil-residing microbial communities and maize simultaneously by ensuring a long-term supply of available nutrient resources. These observations justify reusing cover crop root channels, and this unique strategy could be a viable option for future agricultural practices.

### Microbiological differences in the cover crop variations

The observed significant differences in 16S rRNA gene copy numbers among the tested monocultures and mixtures indicate that cover crops differentially shape bacterial communities in the root zone and bulk soil. Such studies involving cover crops have been reported [79, 80] before, but using our strategy of reusing root channels has not been done previously. The highest copy numbers were found in samples from monocultures and mixtures containing *Brassicaceae* and *Fabaceae*, with community richness being highest in subsoil samples from plots where these cover crops were grown (showing only insignificant differences among topsoil samples across the variants). *Brassicaceae* oilseed radish has a deep-reaching tap root, and *Fabaceae* red clover has a deep root system, both of which support diverse microbial metabolic activity and growth in the subsoil. The subsoil-reaching root zones of these cover crops have comparatively more active metabolic hotspots as compared to fallow [110]. When maize reuses these root channels, it can exploit the nutrient resources from the deeper subsoil niches and escalate metabolic dynamics. For the absolute abundances of bacterial communities and their structure, we hypothesize that along the spatiotemporal parameters, microbial metabolic plasticity comes into effect and does not alter the community frameworks significantly [111] (Fig. 6A and B). For instance, glycolytic enzymes from *Chloroflexota* dominate in the topsoil and during VE, whereas enzymes involved in the pentose phosphate pathway are more prevalent in the subsoil and during R1–RX. *Bacteroidota* dominates the pentose phosphate pathway roles during VE and *Pseudomonadota* during V1–VX. This indicates a role-sharing plasticity among the communities without affecting their structural framework.

In samples from maize root zone soil in cover crop root channels, we detected an increase in the number of proteins involved in the C and N cycles along the maize growth stages. The activities of glycolysis and the pentose phosphate pathway differed significantly among the variations, with the cover crop mixtures showing higher expression of enzymes than the monocultures. The glycolytic enzymes enolase, GAPDH, phosphoglycerate mutase, and PK were differentially abundant in the root zone of each variation. Notably, their abundance was lower in profiles from *Fabaceae* samples compared to *Brassicaceae* and *Poaceae*, indicating that the flux of organic C into the soil was smaller with *Fabaceae*. This is consistent with the role of *Fabaceae* in facilitating nitrogen fixation, which requires a substantial portion of the available metabolic energy [112]. Interestingly, the two mixtures of *Brassicaceae*/*Fabaceae* and *Poaceae*/*Fabaceae* had the overall highest relative abundance levels of these proteins. These results corresponded to an increase in the abundance of bacteria contributing to several steps of the C cycle in the profiles with cover crop mixtures, providing initial evidence for a synergistic effect of cover crop mixtures on bacterial C cycle dynamics in the maize root zone.

There was evidence of different N cycle dynamics in the maize root zone following the use of various cover crops. However, the findings were not as conclusive as those for the C cycle; partly several enzymes expected to be present in the samples such as ammonium monooxygenase (AmoA) and dissimilatory nitrite reductase (NirK/S) were not detected. In general, proteomics may be less sensitive than the nucleic acid–based analyses qPCR and PCR amplicon sequencing because it does not include an amplification step. Nonetheless, some proteome data showed higher abundances of dissimilatory NAR and NXR in samples from *Fabaceae* and *Poaceae* monocultures and mixtures, indicating enhanced N-cycle dynamics with these cover crops. Additionally, glutamine synthetase and urease were more abundant in samples from *Fabaceae* and *Poaceae* monocultures and mixtures, suggesting greater N utilization efficiency in the root zone of these cover crops. Improved N-cycle efficiency and dynamics by the reusage of cover crop root channels could lead to better management of nitrogen resources by cash crops in the soil regimes. Further studies investigating the reusage of root channels could provide additional insights into the observed functional dynamics in the soil profile and involved microbes. We would be able to extract more data about the changes influenced by different cover crop variations on metabolic pathway dynamics in soils having different compositions under various environmental conditions such as heat stress and water stress.

### Limitations of a complex system like the soil root zone

The combination of amplicon sequencing– and metaproteomics-based approaches to study the functional dynamics of microbial communities in the soil root zone is innovative and informative. However, there are still challenges in extracting the optimal output from these techniques. Due to the dynamics of the C and N cycles and the difficulties of extracting proteins from soil samples, some enzymes with key roles, especially in the subsoil, are often missed. This limitation prevents us from visualizing the complete picture of microbial involvements and interactions in metabolic cycles in the soil profiles. Nevertheless, our approach allowed us to obtain a substantial amount of protein groups to generate a functional outlook of key microbial players in both the topsoil and subsoil. Linking amplicon sequencing studies with metaproteomics requires further improvement due to deficiencies and sensitivities in the available datasets. However, this is expected to improve with the expansion of available datasets. Additionally, integrating information about the combined root network structures, crop yields, soil properties, water and nutrient balance, and carbon sequestration in the soil (especially subsoil) in different climate conditions and extremities would be beneficial. This integration would enhance our understanding of the functional processes and key microbial players after reusing the root channels.

## Conclusion

The reuse of cover crop root channels increased the overall bacterial abundance in the maize root zone, with the highest abundances occurring after cover crop mixtures, according to qPCR and metaproteomics results. The highest bacterial abundances in the subsoil were found with the deep-rooting cover crops red clover (*Fabaceae*) and oilseed radish (*Brassicaceae*), both when grown as monoculture and as part of a mixture, with the deep-rooting fescue (*Poaceae*) having no such effect on the abundance in the subsoil. Furthermore, the bacterial community and the detected proteins of each phylum differed as a function of spatiotemporal parameters and cover crop variations, illustrating the dynamics of the root zone. Mixtures, especially of *Fabaceae*, *Brassicaceae*, and *Poaceae*, increased the abundance of enzymes involved in the C and N cycles. Enzymes of the N cycle were higher in abundance in the presence of *Fabaceae*, while the abundance of C-cycle enzymes was the highest with *Brassicaceae* and *Poaceae*. By directly linking the taxonomic profiles and functional traits, we were able to illuminate the framework of the bacterial communities in the root zone in the top- and subsoil and to delineate dynamics in the microbiome of reused cover crop root channels for maize growth stages. Such information will be beneficial in the selection of cover crop species for root channel reuse and will ultimately support knowledge-based strategies for agricultural practices from a microbiological and biochemical perspective.

## Supplementary Material

Supplementary_Figures_ycae132

Supplementary_Figure_S5_heatmap_C_cycle_extended_ycae132

Supplementary_Figure_S6_heatmap_N_cycle_extended_ycae132

Supplementary_Tables_S1_S12_ycae132

## Data Availability

The authors declare that the data supporting the findings of this study are available within the article and its supplementary information files, and from the corresponding authors on request. The metaproteomics datasets generated during the current study are available in the PRIDE data repository with the sample metadata, vide PRIDE dataset identifier PXD046832 (https://www.ebi.ac.uk/pride). The raw sequencing data and the respective metadata generated from this study are available under NCBI BioProject ID PRJNA1045860, which can be accessed using the link: https://www.ncbi.nlm.nih.gov/sra/PRJNA1045860. The raw qPCR data along with sample metadata are available on *Zenodo* under the DOI: https://doi.org/10.5281/zenodo.10061192.
