# Supplementary material for: Cover crop monocultures and mixtures enhance bacterial abundance and functionality in the maize root zone
Source: ISME Commun. 2024 Oct 29;4(1):ycae132. doi: 10.1093/ismeco/ycae132 (PMC11546721; doi:10.1093/ismeco/ycae132)
Supplement: Supplementary_Figures_ycae132 [file supplementary_figures_ycae132.docx]

Appendix A. Supplementary data

Cover crop monocultures and mixtures enhance

bacterial abundance and functionality in the maize root zone

Ghosh et al.

# Supplementary Figures

# Figure S1: An alluvial flowchart representing the different variations and the identified bacterial phyla in each one of them.


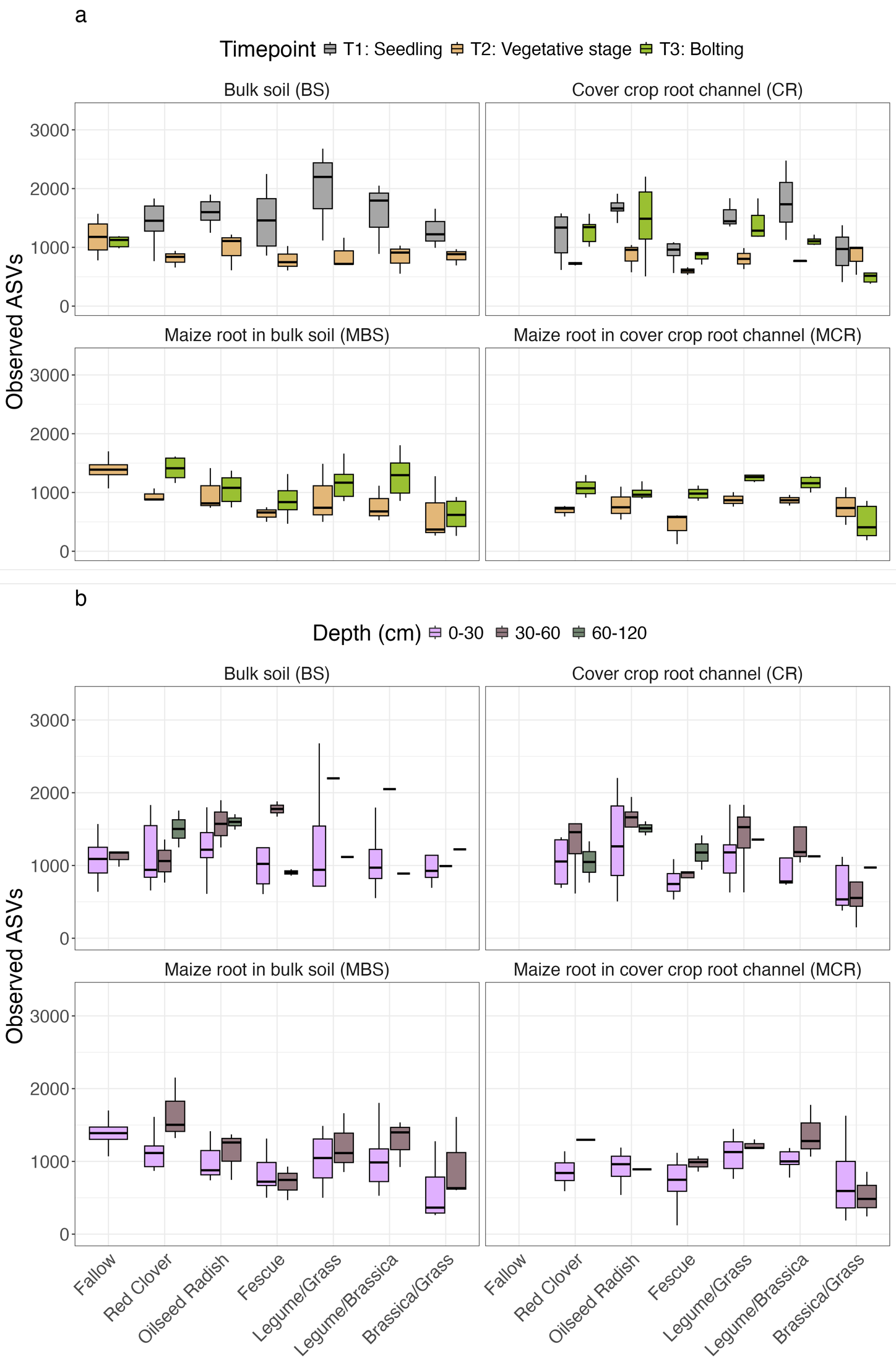


Figure S2: **a, b.** Observed amplified sequence variants (ASVs) depicting phylotype richness for the variations of cover crops over the growth phases of maize and depths of the soil horizon, with the different soil sample sources segmented separately (BS, CR, MBS, MCR). The time points taken into consideration are T_1_: seedling (VE), T_2_: vegetative stage (V1-VX), and T_3_: bolting (R1-RX). The depths from which the soil samples were collected are topsoil (0–30 cm) and subsoil (30–60 cm, 60–120 cm). For T_1_, *n* = 56; for T_2_, *n* = 73; for T_3_, *n* = 108.


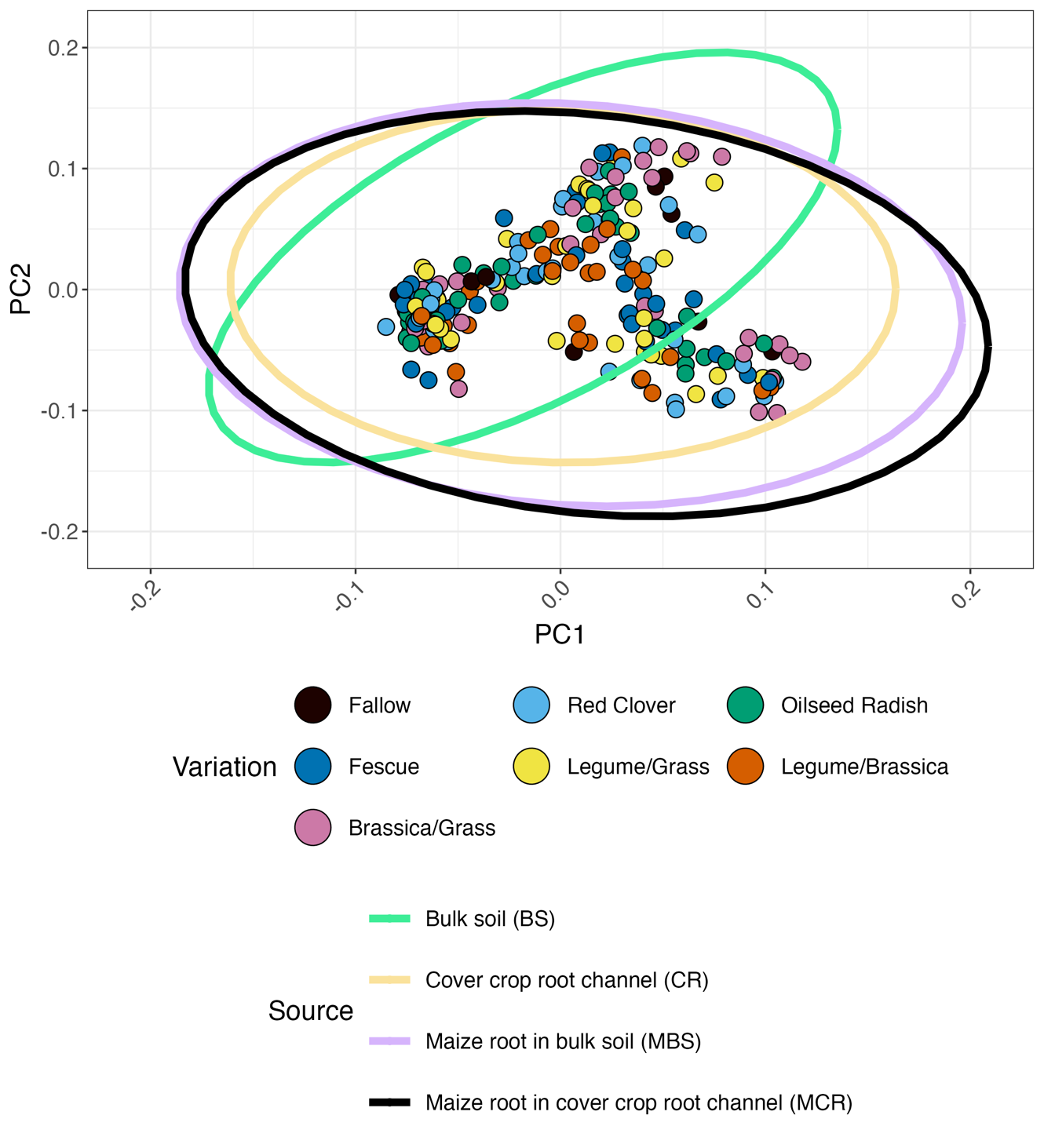


Figure S3: Bacterial community beta-diversity visualized using PCoA ordination based on weighted UniFrac distances for the different cover crop variations along the different sample sources (BS, CR, MBS, and MCR) (*n* = 237).


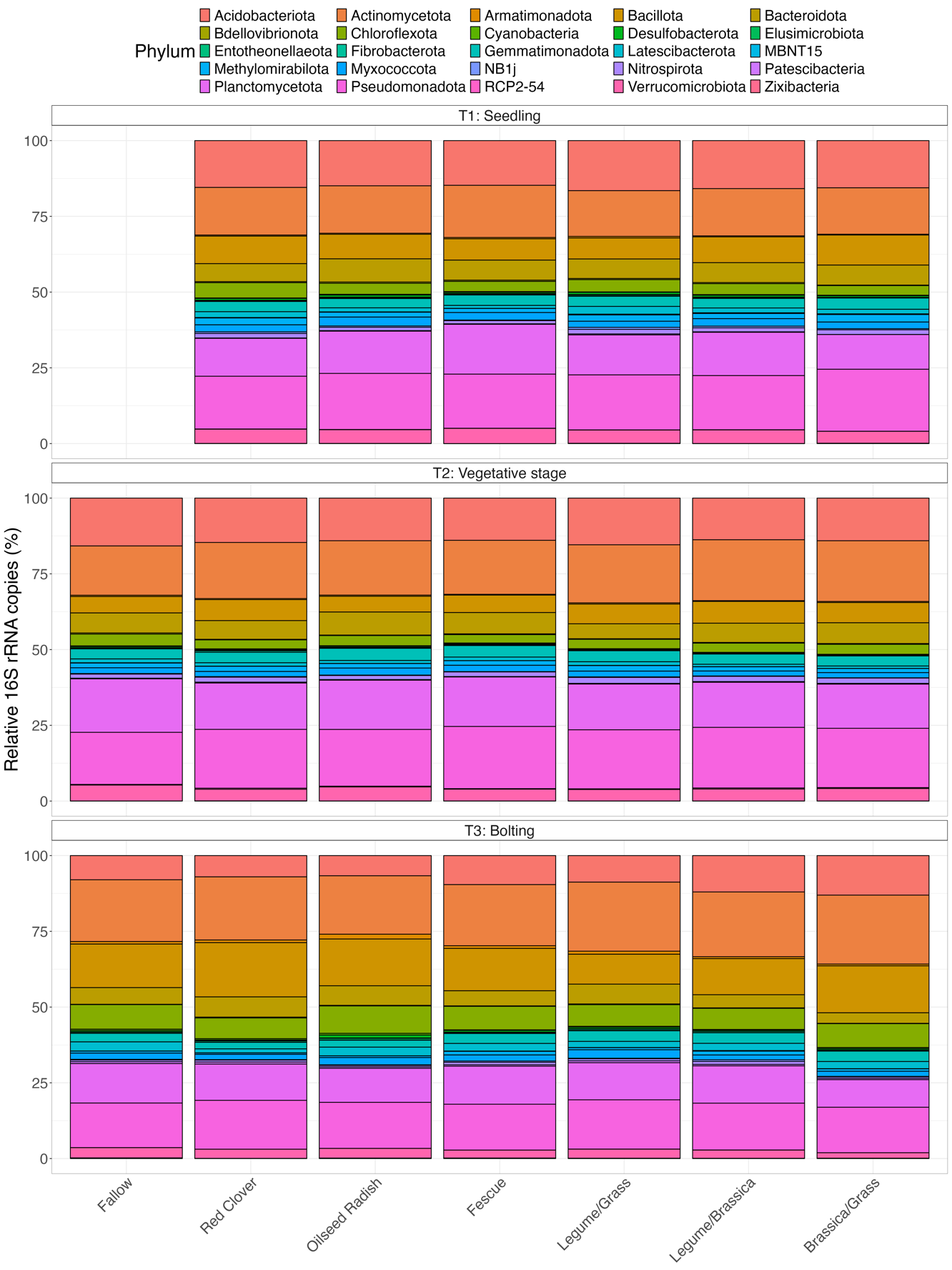


Figure S4: Relative abundance of the top twenty-five taxa in the distinct variations and maize growth phase time points, as measured by 16S rRNA gene amplicon sequencing, followed by quantification with quantitative PCR (qPCR). T_1_: seedling (VE), T_2_: vegetative stage (V1-VX), and T_3_: bolting (R1-RX). The number of samples used for the estimation – for T1, *n* = 56; for T2, *n* = 73; for T3, *n* = 108.

Figure attached as a vector file (Supplementary Figure S5, PDF)

Figure S5: The extended C-cycle heatmap from metaproteomics, which includes all the enzymes heavily or sparsely involved in the different steps of the C cycle and bacterial phyla corresponding to the enzymes. For proteomics, *n* = 232; time point parameters - T_1_: seedling (VE), T_2_: vegetative stage (V1-VX), and T_3_: bolting (R1-RX). (Additional vector file provided for detailed observations)

Figure attached as a vector file (Supplementary Figure S6, PDF)

Figure S6: The extended N-cycle heatmap from metaproteomics, which includes all the enzymes heavily or sparsely involved in the different steps of the N cycle and bacterial phyla corresponding to the enzymes. For proteomics, *n* = 232; time point parameters - T_1_: seedling (VE), T_2_: vegetative stage (V1-VX), and T_3_: bolting (R1-RX). (Additional vector file provided for detailed observations)


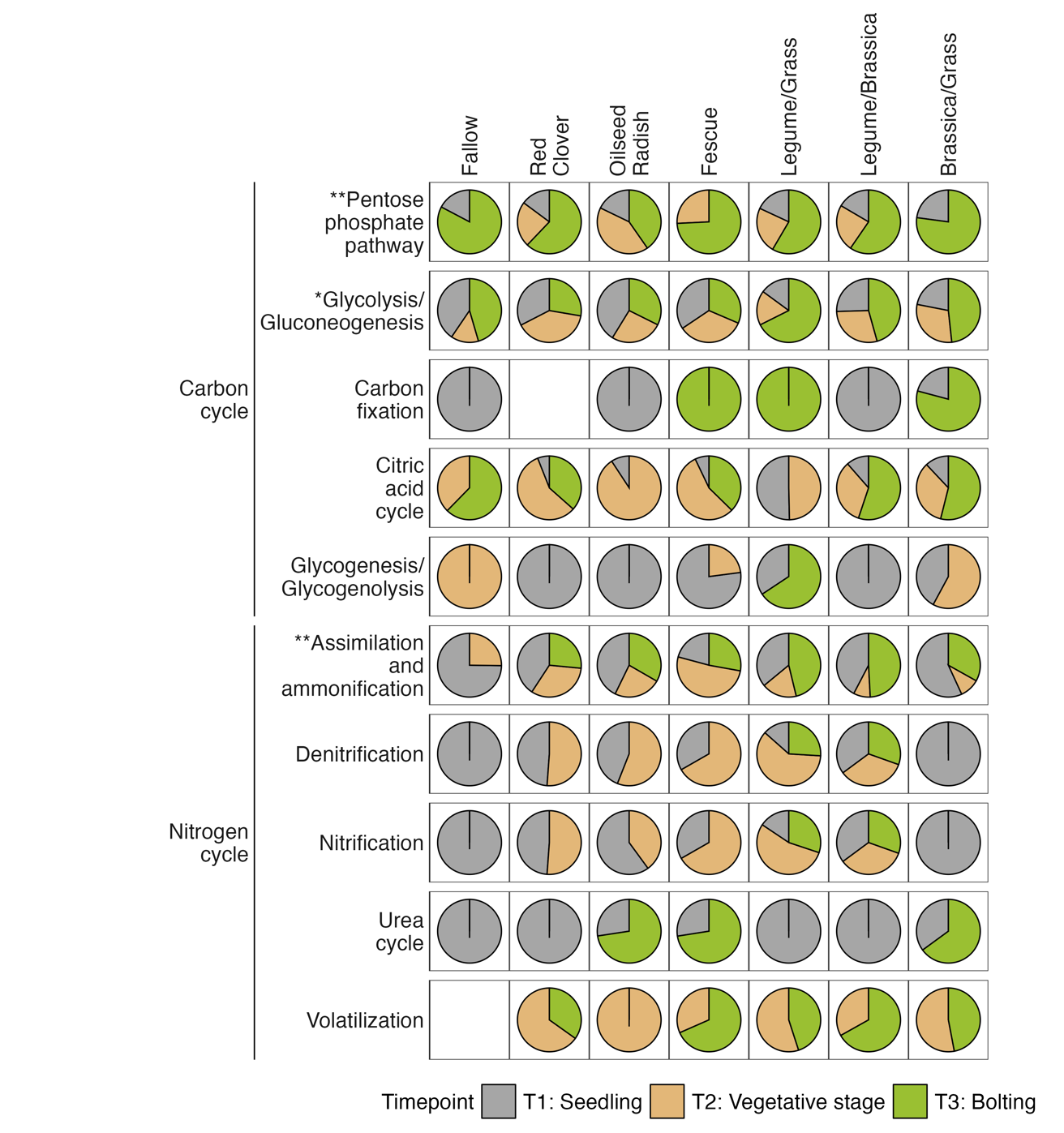


Figure S7: A trend for the expression of the different steps of the C and N cycle along stages of maize growth. The pie-chart measurements were calculated as a percentage of the overall expression of enzymes in all the steps involved in the C and N cycles, for each soil sample variation, and maize growth stages. The empty spaces indicate no identification of enzymes involved in the steps from that specific phyla, as observed in the metaproteomic analysis. The stars with the names of phyla represent significant differences concerning the different variations. **p* < 0.05, ***p* < 0.01, ****p* < 0.001. T_1_: seedling (VE), T_2_: vegetative stage (V1-VX), and T_3_: bolting (R1-RX) (Supplementary Table S9c provides the data for pairwise test for significance for the steps of the C and the N cycle along the parameters of variations and maize growth stages)


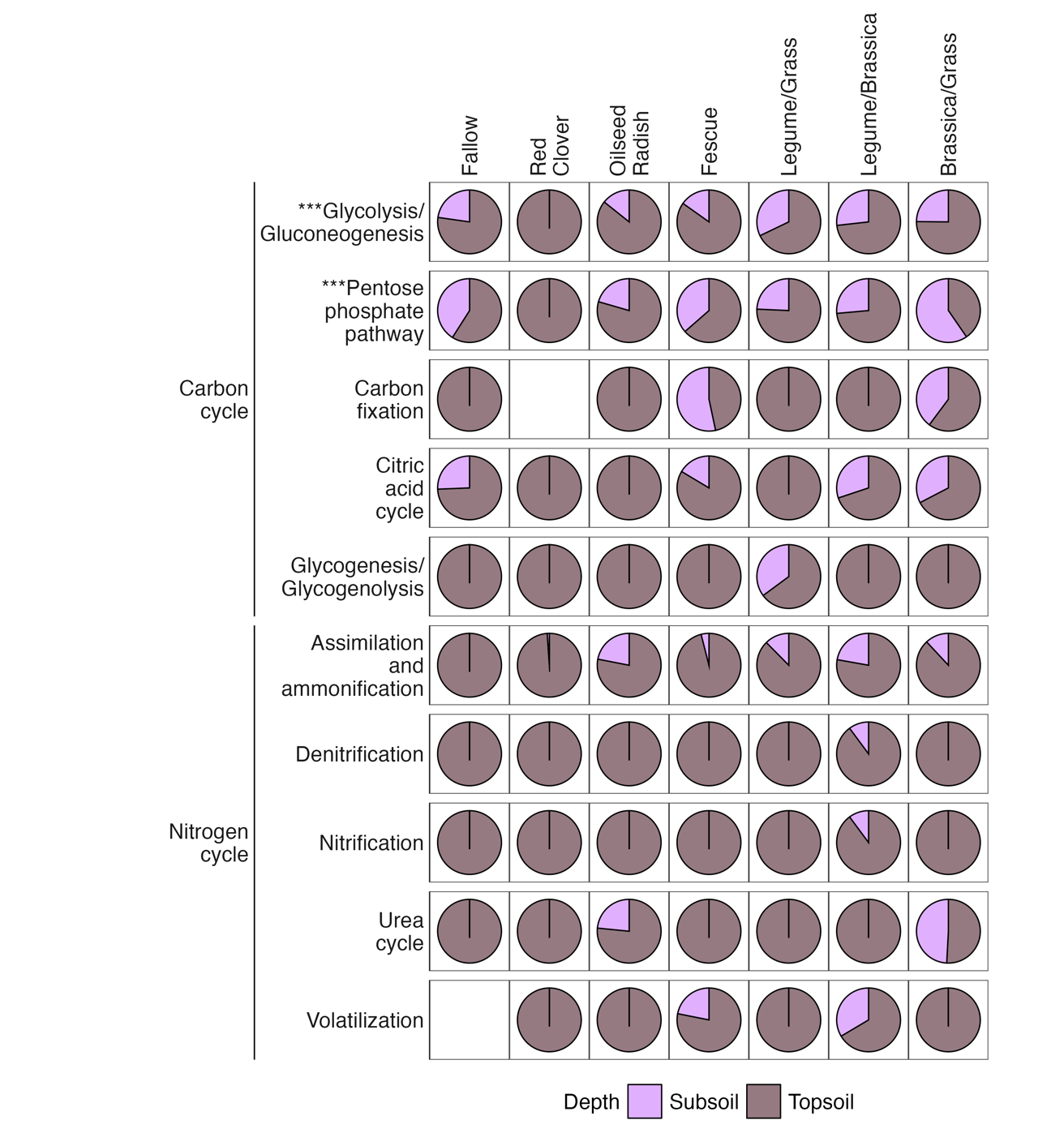


Figure S8: A trend for the expression of the different steps of the C and N cycle along the depth of soil profiles. The pie-chart measurements were calculated as a percentage of the overall expression of enzymes in all the steps involved in the C and N cycles, for each soil sample variation, and rhizosphere horizon depths. The empty spaces indicate no identification of enzymes involved in the steps from that specific phyla, as observed in the metaproteomic analysis. The stars with the names of phyla represent significant differences concerning the different variations. Topsoil: 0-30 cm, Subsoil: 30-60 cm; **p* < 0.05, ***p* < 0.01, ****p* < 0.001. (Supplementary Table S9d provides the data for pairwise test for significance for the steps of the C and the N cycle along the parameters of variations and depths)


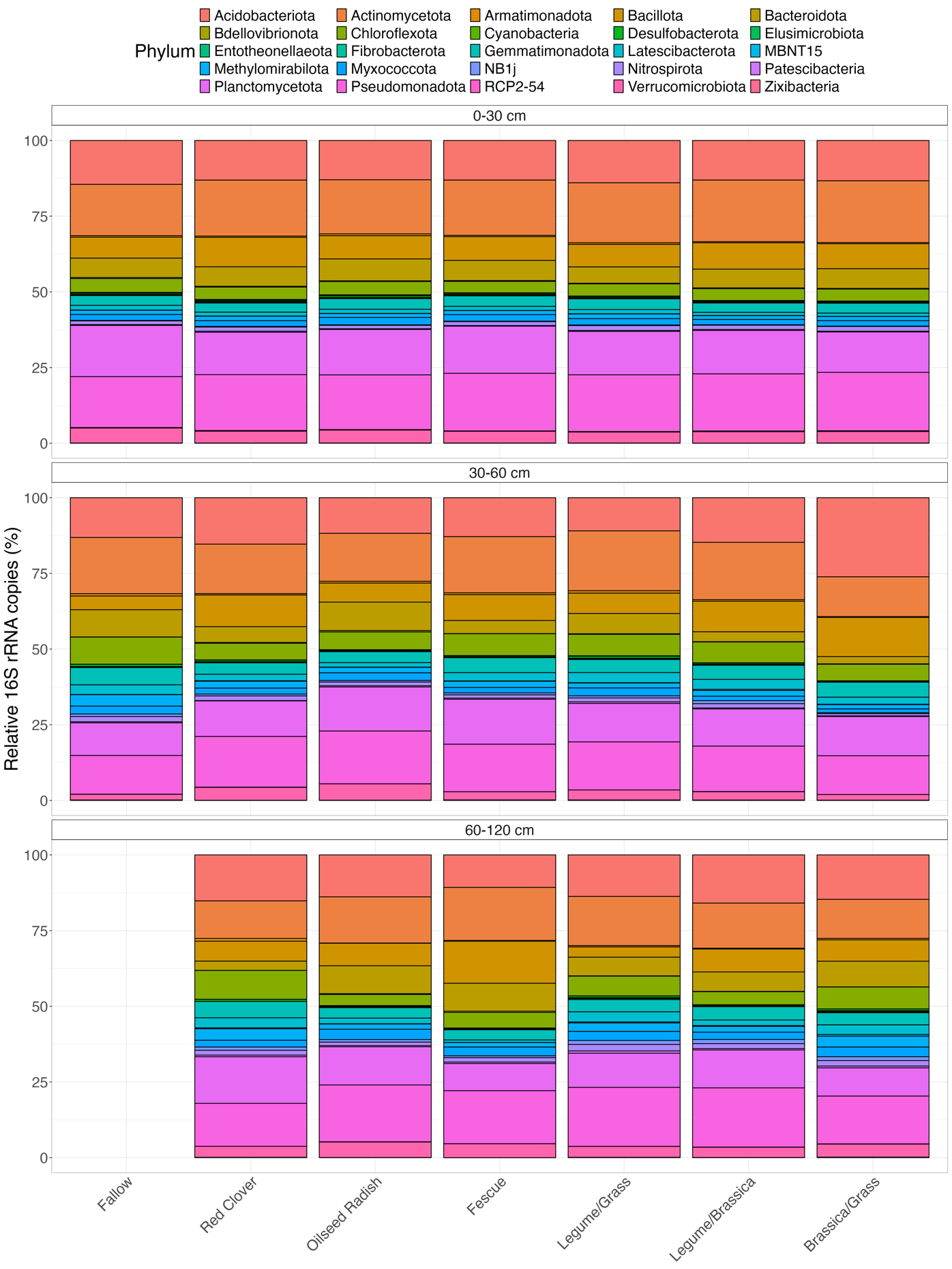


Figure S9: Relative abundance of the top twenty-five taxa in the distinct variations and the soil horizon depths, as measured by 16S rRNA gene amplicon sequencing, followed by quantification with quantitative PCR (qPCR). The horizontal depth of the soil for the collected samples are topsoil (0-30 cm), and subsoil (30-60 cm, 60-120 cm) (*n* = 237).
