# Supplementary material for: Cover crop monocultures and mixtures enhance bacterial abundance and functionality in the maize root zone
Source: ISME Commun. 2024 Oct 29;4(1):ycae132. doi: 10.1093/ismeco/ycae132 (PMC11546721; doi:10.1093/ismeco/ycae132)
Supplement: Supplementary_Figure_S5_heatmap_C_cycle_extended_ycae132 [file supplementary_figure_s5_heatmap_c_cycle_extended_ycae132.pdf]

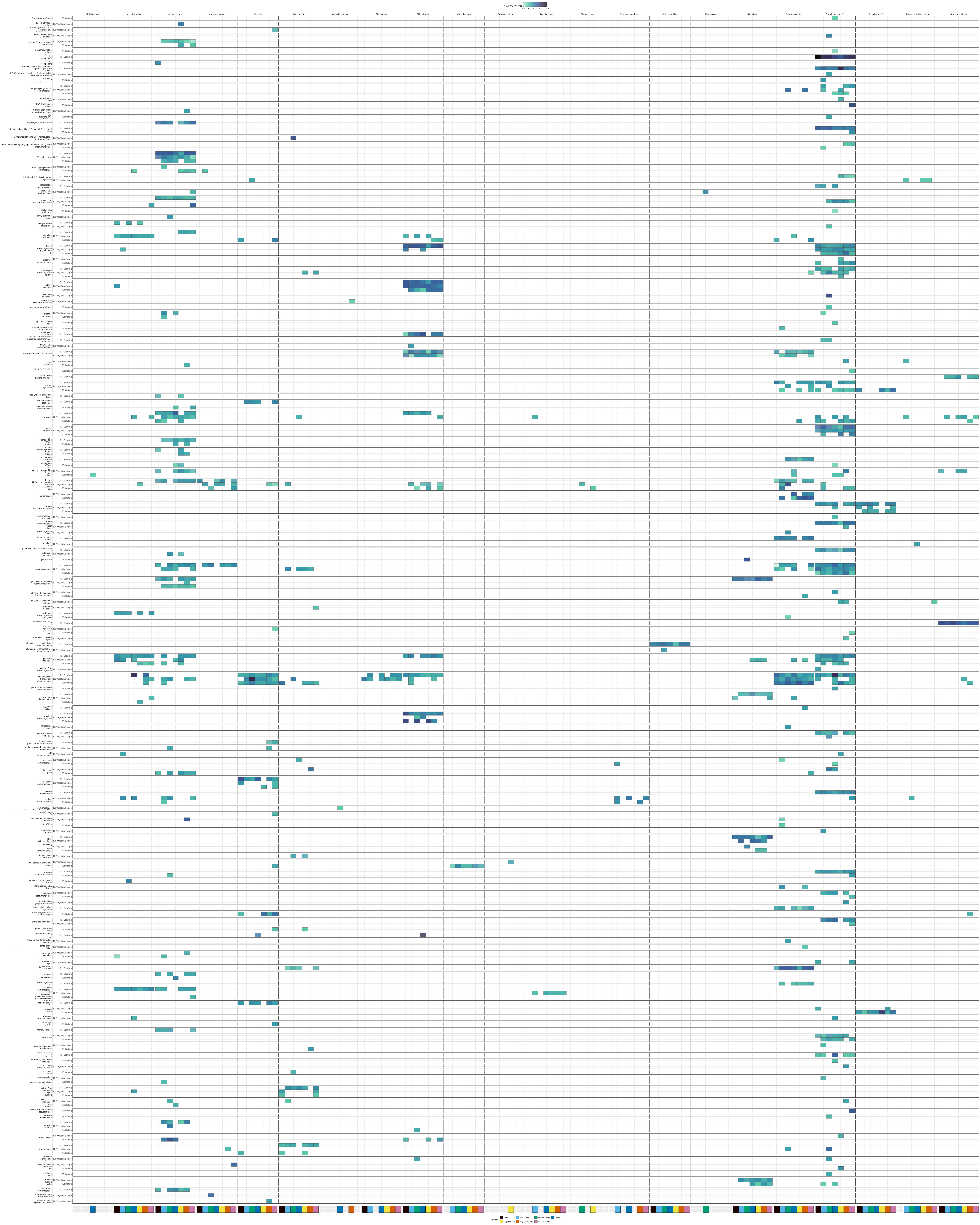
log2 [LFQ intensity]
7.5
10.0
12.5
15.0
17.5
Abditibacteriota
Acidobacteriota
Actinomycetota*
Armatimonadota
Bacillota
Bacteroidota
Campilobacterota
Chlamydiota
Chloroflexota
Cyanobacteria
Cyanobacteriota
Dadabacteria
Fibrobacterota
Gemmatimonadota
Methylomirabilota
Myxococcota
Nitrospirota*
Planctomycetota*
Pseudomonadota**
Spirochaetota***
Thermodesulfobacteriota
Verrucomicrobiota
T1: Seedling
T2: Vegetative stage
T3: Bolting
Variation
Fallow
Red Clover
Oilseed Radish
Fescue
Legume/Grass
Legume/Brassica
Brassica/Grass
